# Histone Methyltransferase G9a Promotes the Development of Renal Cancer through Epigenetic Silencing of Tumor Suppressor Gene SPINK5

**DOI:** 10.1155/2021/6650781

**Published:** 2021-07-13

**Authors:** Ren-Gui Li, Huan Deng, Xiu-Heng Liu, Zhi-yuan Chen, Shan-shan Wan, Lei Wang

**Affiliations:** ^1^Department of Urology, Renmin Hospital of Wuhan University, Wuhan, China; ^2^Chongqing University Cancer Hospital, Chongqing, China; ^3^Department of Gastroenterology, Renmin Hospital of Wuhan University, Wuhan, China; ^4^Department of Ophthalmology, Renmin Hospital of Wuhan University, Wuhan, China

## Abstract

**Background:**

Renal cell carcinoma (RCC) accounts for approximately 2–3% of malignant tumors in adults, while clear cell renal cell carcinoma accounts for 70–85% of kidney cancer cases, with an increasing incidence worldwide. G9a is the second histone methyltransferase found in mammals, catalyzing lysine and histone methylation. It regulates gene transcription by catalyzing histone methylation and interacting with transcription factors to alter the tightness of histone-DNA binding. The main purpose of this study is to explore the role and mechanism of G9a in renal cell carcinoma.

**Methods:**

Firstly, we investigated the expression of G9a in 80 clinical tissues and four cell lines. Then, we explored the effect of G9a-specific inhibitor UNC0638 on proliferation, apoptosis, migration, and invasion of two renal cancer cell lines (786-O, SN12C). In order to study the specific mechanism, G9a knocking down renal cancer cell line was constructed by lentivirus. Finally, we identified the downstream target genes of G9a using ChIP experiments and rescue experiments.

**Results:**

The results showed that the specific G9a inhibitor UNC0638 significantly inhibited the proliferation, migration, and invasion of kidney cancer *in vivo* and *in vitro*; similar results were obtained after knocking down G9a. Meanwhile, we demonstrated that SPINK5 was one of the downstream target genes of G9a through ChIP assay and proved that G9a downregulate the expression of SPINK5 by methylation of H3K9me2. Therefore, targeting G9a might be a new approach to the treatment of kidney cancer.

**Conclusion:**

G9a was upregulated in renal cancer and could promote the development of renal cancer *in vitro* and *in vivo*. Furthermore, we identified SPINK5 as one of the downstream target genes of G9a. Therefore, targeting G9a might be a new treatment for kidney cancer.

## 1. Introduction

Renal cell carcinoma (RCC) accounts for approximately 2–3% of malignant tumors in adults, while clear cell RCC accounts for 70–85% of kidney cancer cases, with an increasing incidence worldwide [1, 2]. Clinically, RCC is divided into four stages according to the size of tumor and extent of invasion and metastasis [3]. The occurrence of tumors has been previously related with mutations in tumor suppressor genes and/or protooncogenes. Recently, numerous studies have shown that epigenetic regulation mechanisms of gene expression, such as DNA and histone modification abnormalities, play an important role in tumorigenesis. Histone methyltransferases (HMTs) are a class of S-adenosylmethionine as a methyl donor that catalyzes the transfer of 1–3 methyl groups to histones [4]. G9a is the histone lysine 9 (H3K9) methyltransferase [5, 6], and H3K9 methylation is involved in the formation of heterochromatin, DNA methylation, and transcriptional silencing, which are related to the development of cancer [7, 8].

G9a consists of a SET catalytic domain, an ankyrin repeat, and a glutamate- and cysteine-rich region at the N-terminus [9]. The SET domain is located at the carboxy terminus of G9a and is a classical HMT catalytic domain responsible for the addition of methyl groups on histone H3 [10]. G9a is the second HMT to be discovered in mammals, catalyzing lysine methylation [11] and histone methylation [12]. Histones are an important component of chromatin. G9a regulates gene transcription by catalyzing histone methylation and interacting with transcription factors to alter the tightness of histone-DNA binding [13]. A previous study showed that G9a could mainly catalyze the methylation of H3K9 (H3K9me2) through the addition of 1–3 methyl groups. Cells with abnormal epigenetic modifications have been found in various tumors, such as breast cancer [14], lung cancer [15], head and neck cancer [16], and ovarian cancer [17]. G9a has been the focus recently because it plays an important role in promoting tumorigenesis and metastasis [18, 19]. However, its mechanism in RCC still remains unclear.

The main purpose of this study was to investigate the mechanism of G9a in RCC. In this study, the specific inhibitor of G9a UNC0638 [20] was used to inhibit G9a expression and demonstrated its effects on the proliferation, apoptosis, migration, and invasion of RCC. UNC0638 has greater than 10,000-fold selectivity to SET7/9 (a H3K4 HMTase), SET8 (a H4K20 HMTase), PRMT3, and SUV39H2. In MDA-MB-231 cells, UNC0638 reduced H3K9me2 levels in a concentration-dependent manner with IC50 of 81 nM. UNC0638 treatment of various cell lines resulted in a lower global H3K9me2 level reduction, which is consistent with the effect observed with shRNA to reduce G9a and GLP. Furthermore, the targeted genes of G9a were also explored and verified *in vivo* and *in vitro*.

## 2. Materials and Methods

### 2.1. Cell Culture

Human RCC cell lines (786-O, SN12C, and OSRC-2) and renal tubular epithelial cells (HK-2) were afforded by the China Center for Type Culture Collection. SN12C cells were cultured in Dulbecco's modified Eagle's medium (HyClone), and 786-O cells were cultured in RPMI-1640, both supplemented with 10% fetal bovine serum (Gibco) and 1% antibiotic solution (penicillin 100 U/ml and streptomycin 100 g/ml) (Beyotime, China). UNC0638 was obtained from Selleck and dissolved in dimethyl sulfoxide at 5 mM for stock solution, which was stored at −20°C. All experiments were performed in mycoplasma-free condition [21].

### 2.2. Bioinformatics

We performed bioinformatics analysis and acquired The Cancer Genome Atlas (TCGA) data from the Human Protein Atlas. We could intuitively observe the expression of G9a in tissues and cells. We used free bioinformatics website oncomine to analyze G9a expression in human kidney cancer. After being lodged on, four datasets including G9a expression data obtained from Shankavaram Cell Line [22], Compendia Cell Line (Not Published 2007/11/14), Staunton Cell Line [23], and Shankavaram Cell Line 2 [22] were induced. After data standardization, expression data was presented in the five CRC cell lines as mean ± standard deviation (SD).

### 2.3. Cell Proliferation Assay

Cell Counting Kit-8 (CCK-8), purchased from Beyotime, was used to evaluate cell viability. RCC cells were seeded into 96-well plates (7 × 10^3^/plate) overnight. Then, CCK-8 solution was added into each well for 2 h at 37°C. Microplate reader (Victor3 1420 Multilabel Counter; PerkinElmer) was used to measure the absorbance at 450 nm.

### 2.4. Western Blotting

RCC cells were cultured into a six-well plate for 24 h and then subjected to different treatments. Protein concentration was measured by Bicinchoninic Acid Kit (Beyotime). Then, proteins were added to sodium dodecyl sulfate-polyacrylamide gel for electrophoresis and then transferred to polyvinylidene difluoride membranes (Millipore). After being washed three times, the membranes were blocked with 5% nonfat dry milk in tris-buffered saline (TBS). Primary antibodies were incubated overnight at 4°C. After being washed, secondary antibodies were incubated for 1 h at room temperature. Finally, the membranes were scanned using a two-color Odyssey infrared imaging system (LI-COR Biosciences). Protein bands were quantified by densitometry using the ImageJ software (National Institutes of Health, Bethesda, MD, USA) [21].

### 2.5. Colony Formation Assay

500 cells per well were seeded to six-well plates and incubated for one week. The cell colonies were fixed with 4% paraformaldehyde and stained with 0.1% crystal violet solution. Cell colonies (>50 cells) were counted.

### 2.6. Flow Cytometry for Apoptosis

The Annexin V-PE/7-AAD kit (MultiSciences) and Annexin-V-FITC/PI kit (BD Biosciences) were used to calculate the number of apoptotic cells by flow cytometry (FACSCalibur; Becton Dickinson). All cells were cultured into six-well plates, then incubated for 24 h with different treatments. Adherent cells were collected and costained with 5 *μ*l Annexin V-PE and 5 *μ*l 7-AAD prior to flow cytometric analysis.

### 2.7. Wound Healing Assay

786-O and SN12C cells were cultured in six-well plates. Then, a wound was scratched on the cell surface by the tube. After different treatments, the cells were incubated in serum-free medium for different times. Finally, five random areas were taken from each well to observe the migratory ability through a microscope (BX51; Olympus Corporation, Tokyo, Japan).

### 2.8. Transwell Assay

The cells were digested with trypsin and diluted to 1 × 10^5^/ml in Dulbecco's modified Eagle's medium (containing 1% FBS). Then, 600 *μ*l medium with 20% FBS was added into lower chambers of the transwell system. Matrigel (BD Biosciences) was coated on the upper chambers. After 80 *μ*l cell suspensions were added, the plates were incubated at 37°C for 24 h. The cells, which invaded into the lower layer, were washed with phosphate-buffered saline, fixed with methanol, and stained with 1% crystal violet. Invasive cells were counted in five random fields for each well by an inverted microscope (BX51; Olympus Corporation) [21].

### 2.9. Establishment of G9a and Serine Protease Inhibitor Kazal-Type 5 (SPINK5) Knockdown Cell Lines

The G9a and SPINK5 stable knockdown cell lines were established through the overexpression of lentiviral-based stable short hairpin RNA (shRNA). Nontarget shRNA was obtained from Sigma–Aldrich as negative control. The lentiviral vector was GV248, and the targeted sequence for G9a was GGGTCACTTCTCCTGAACGC. The sequence of the small interfering RNA (shRNA) for SPINK5 was GCTGAAGCACGAGCTAGAA. The effect of G9a and SPINK 5 knockdown was validated through western blotting.

### 2.10. Chromatin Immunoprecipitation (ChIP) Assay

ChIP was applied to analyze the interaction of G9a and H3K9me2 methyl transferase and SPINK5 gene. Immunoglobulin G was used as internal control. A specific primer (TTAGAGTAGTTGGGATTATAGGTGC) of the promoter sequence of SPINK5 was designed and synthesized. Quantitative polymerase chain reaction (qPCR) was used to detect the level of SPINK5.

### 2.11. Xenograft Tumor Experiment in Nude Mice

The animal experiments were approved by the Ethics Committee of Renmin Hospital, Wuhan University (Wuhan, China). Male BALB/c nude mice (5 weeks old) were purchased from Beijing Life River Experimental Animal Technology Co. Ltd. (Beijing, China). The collected 786-O cells were washed in serum-free RMPI-1640, suspended in 100 *μ*l phosphate-buffered saline, and subcutaneously implanted into the dorsal region of nude mice. When the tumor volume reached 100–150 mm^3^, nude mice were randomly divided into four groups (*n* = 5 per group), namely, the normal group, negative control group, UNC0638 group, and G9a knockdown group. Mice in the UNC0638 group were intraperitoneally injected (5 mg/kg) once every 48 h. Tumor size was measured using a Vernier caliper (3–4 times per week). Tumor volume (mm^3^) was calculated as follows: 0.5 × *d*^2^ × *D*, as *d* and *D* were the shortest and longest diameter, respectively. All mice were weighed 3–4 times per week. After 30 days, tumor specimens were collected for further experiments.

### 2.12. Immunohistochemistry Staining

The 4 *μ*m paraffin sections were deparaffinized, hydrated, and microwave-repaired. Endogenous peroxidase was removed using 3% hydrogen peroxide, and the primary antibodies were added and incubated overnight at 4°C. Then, secondary antibodies were incubated for 1 h at room temperature. And horseradish peroxidase was added to react with 3,30-diaminobenzidine.

### 2.13. Ethics Approval and Consent to Participate

All experiments were approved by the Ethics Committee of Renmin Hospital, Wuhan University (Wuhan, China), in accordance with the ethical standards of institutions and/or national research committees, the 1964 Helsinki Declaration and its amendments, or comparable ethical standards.

### 2.14. Statistical Analysis

The SPSS software version 20.0 (IBM Corp., Armonk, NY, USA) was used for all analyses. Data were expressed as mean ± standard deviation. Analysis of variance was used to analyze the differences among groups. Student's *t*-test and chi-square analysis were used to make comparisons between two groups, and one-way ANOVA was used in comparisons among multiple groups. A *P* value smaller than 0.05 was considered statistically significant.

## 3. Results

### 3.1. The Upregulation of G9a in Human RCC

**Figure 1:**
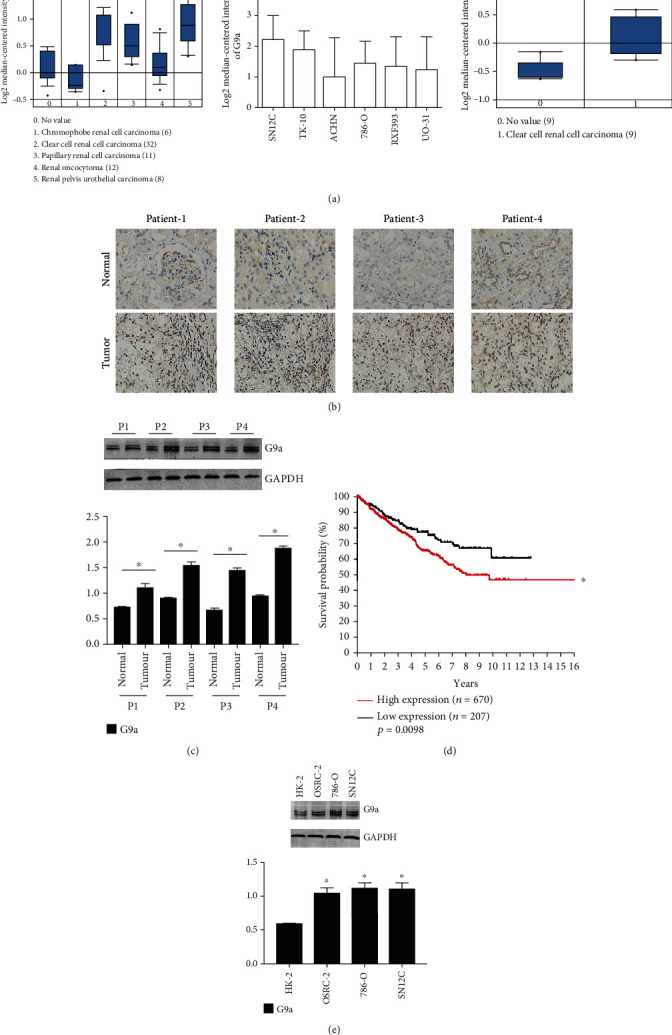
Frequent upregulation of G9a in human RCC. (a) Through bioinformatics data mining, it was revealed that G9a was highly expressed in human renal cancer cell lines and clinical samples (no value means normal tissue). 1: G9a expression in different renal cell carcinomas; 2: G9a expression in different renal cell carcinoma cell lines; 3: G9a expression in clear renal cell carcinoma and normal tissue. (b) G9a expression was significantly upregulated in tumor tissues of most patients with RCC compared with adjacent tissues. (c) G9a expression in four patients was measured. (d) High expression of G9a was associated with poor prognosis of renal cancer (*n* = 877, ^∗^*P* < 0.05). (e) G9a expression was significantly upregulated in 3 renal cancer cell lines (786-O, SN12C, and OSRC-2) compared with human renal tubular epithelial cell line (HK-2).

Tumor tissues and adjacent tissues from 80 patients with RCC were obtained to detect the expression of G9a. Firstly, through bioinformatics data mining, it revealed that G9a was highly expressed in human renal cancer cell lines and clinical samples (Figure 1(a)), and immunohistochemistry showed that G9a was significantly elevated in renal tumor tissues compared with adjacent normal tissues (Figure 1(b)). The same results were observed in western blotting (Figure 1(c)). Besides, we compared the expression levels of G9a in tumor tissues and paracancerous tissues obtained from 80 patients and found that the higher expression of G9a was closely related to the higher stage of RCC (Table 1). Through a bioinformatics analysis and The Cancer Genome Atlas (TCGA) data acquired from the Human Protein Atlas (https://www.proteinatlas.org/ENSG00000204371-EHMT2/pathology/renal+cancer#ihc) website, it could be found that high expression of G9a was associated with poor prognosis of renal cancer (Figure 1(d)). We further measured the expression level of G9a in RCC lines. The human renal tubular epithelial cell line hexokinase-2 (HK-2) and three RCC lines (i.e., 786-O, SN12C, and OSRC-2) were used for western blotting, and the results showed that G9a expression in RCC cell lines was higher than that in HK-2 cells (Figure 1(e)). Therefore, our results showed that G9a was highly expressed in RCC, and its high expression was associated with poor prognosis of RCC.

### 3.2. Inhibition of G9a Significantly Inhibits the Proliferation with Simultaneous Induction of Apoptosis

**Figure 2:**
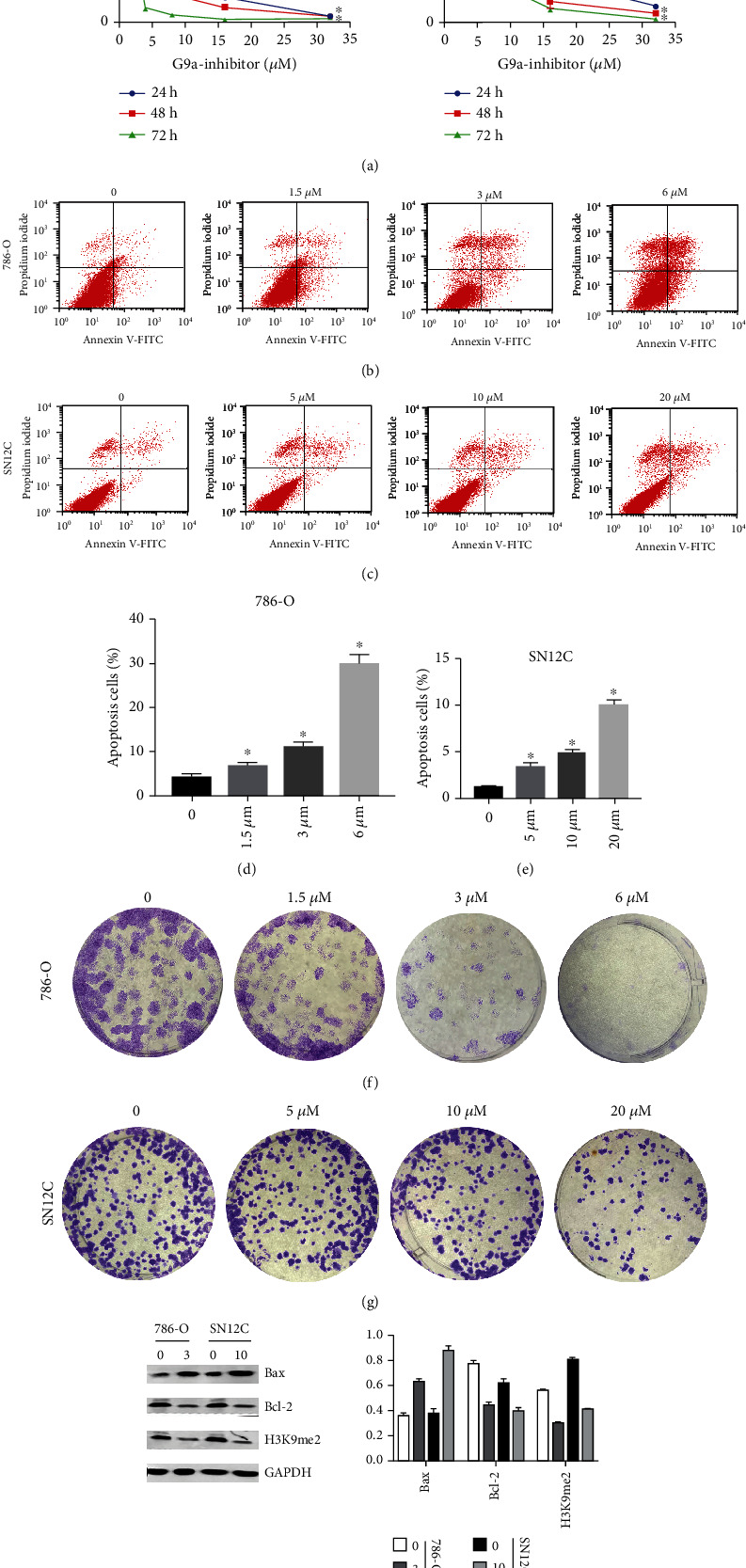
Inhibition of G9a methylation inhibits proliferation with simultaneous induction of apoptosis. (a) 786-O and SN12C cells were treated with different concentrations of UNC0638 (0, 0.5, 1, 2, 4, 8, 16, and 32 *μ*M) for 24 h, 48 h, and 72 h. CCK-8 assay was performed to measure cell growth level. (b–e) 786-O and SN12C were treated with different concentrations of UNC0638 for 24 h and Annexin-V-FITC/PI double-staining assay to detect cell apoptosis through flow cytometry. (f, g) Colony formation experiments were performed to detect how different concentrations of UNC0638 (treated for 24 h) affected proliferation of 786-O and SN12C. (H) The expression of protein-related apoptosis pathway and H3K9me2 was measured by western blot (treated for 24 h).

UNC0638 is a potent, specific inhibitor of G9a that is selective for a broad spectrum of epigenetic and nonepigenetic targets. Treatment of various cell lines with UNC0638 results in lower levels of H3K9me2 [20, 24]. Through the CCK-8 assay, we demonstrated that UNC0638 significantly inhibited the growth of 786-O and SN12C cells (Figure 2(a)). Annexin-V-FITC/PI double-staining assay was used to detect cell apoptosis through flow cytometry. The number of apoptotic 786-O and SN12C cells was gradually elevated as drug concentration increased (Figures 2(b)–2(e)). Similarly, colony formation experiments indicated that the proliferation of 786-O and SN12C cells was significantly inhibited as drug concentrations increased (Figures 2(f) and 2(g)). Besides, western blotting revealed that UNC0638 significantly inhibited H3K9me2 in 786-O and SN12C cells. The expression of the apoptosis gene Bax was elevated when drug concentration increased, whereas the antiapoptosis gene B-cell lymphoma-2 (Bcl-2) decreased (Figure 2(h)). These results suggested that G9a might promote tumor growth and alleviate apoptosis in RCC cells.

### 3.3. Inhibition of G9a Significantly Inhibits Migration and Invasion of RCC Cells

**Figure 3:**
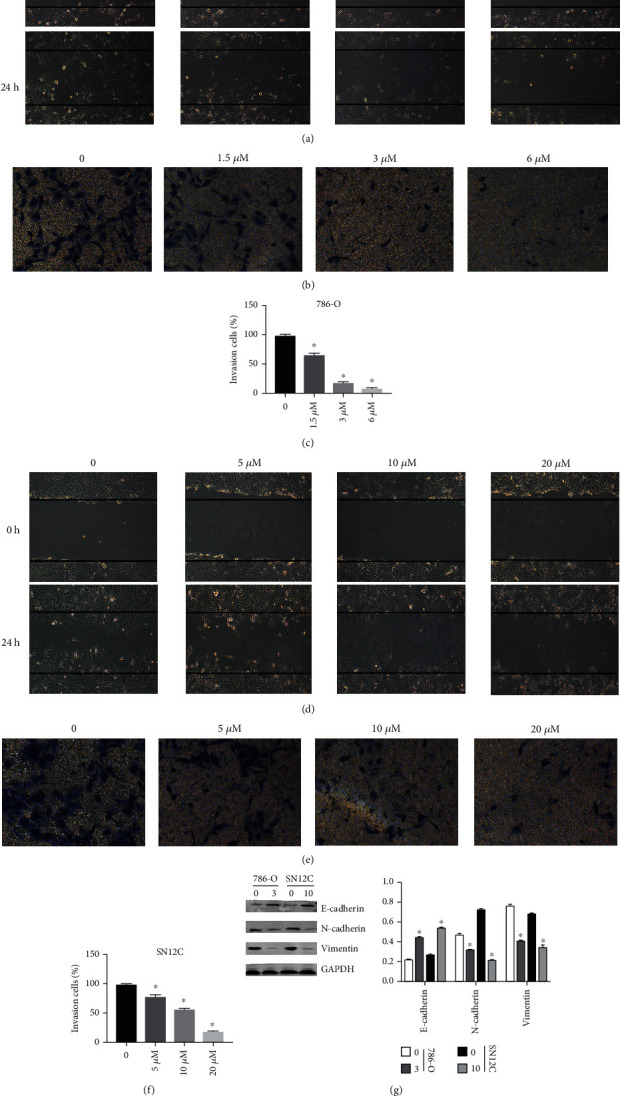
Inhibition of methylation of G9a significantly inhibits migration and invasion of RCC cells. (a) Migratory abilities of 786-O cells were measured through wound healing assay after the addition of inhibitors. (b, c) Invasive abilities of 786-O cells were measured through transwell assay after the addition of inhibitors for 24 h. (d) Migratory abilities of SN12C cells were measured through wound healing assay after the addition of inhibitors. (e, f) Invasive abilities of SN12C cells were measured through transwell assay after the addition of inhibitors for 24 h. (g) The expression of protein-related EMT pathway was measured by western blot.

We performed the wound healing assay and transwell assay to explore the effects of G9a on the migration and invasion of kidney cancer cells. It was found that the migratory and invasive abilities of 786-O cells were significantly inhibited after G9a inhibitor treatment (Figures 3(a)–3(c)). The same result was also observed in SN12C cells (Figures 3(d)–3(f)). Western blotting results suggested that the expression of E-cadherin was significantly increased after treatment with the G9a inhibitor, whereas N-cadherin and vimentin expression decreased (Figure 3(g)). These findings showed that after blocking G9a function, the process of epithelial mesenchymal transformation of tumor cells was significantly inhibited. Thus, G9a might be closely associated with the invasion and metastasis of kidney cancer cells.

### 3.4. Loss of G9a Causes a Decrease in the Carcinogenicity of RCC Cell Lines

**Figure 4:**
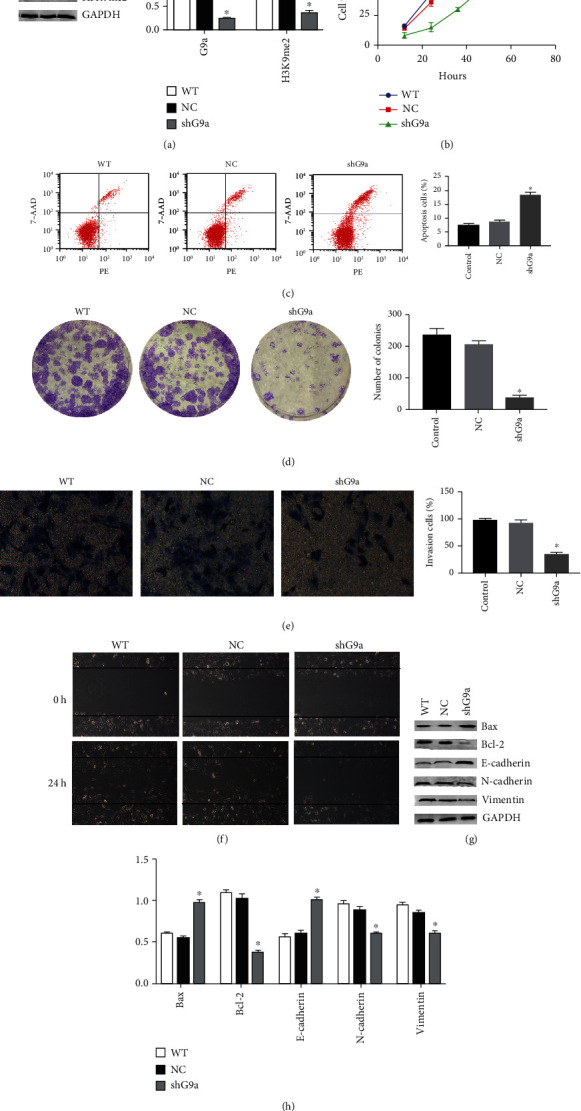
Loss of G9a causes a decrease in the carcinogenicity of RCC cell lines. (a) A stable G9a knockdown RCC line in 786-O was constructed using a lentivirus and expression of G9a, and H3K9me2 was measured by western blot. (b) CCK-8 assay was performed to show the proliferation ability of G9a knockdown RCC line. (c) Detection of apoptosis rate of shG9a-786-O cells via Annexin V-PE/7-AAD double staining (treated for 24 h; WT: wild type; NC: negative control). (d) Colony formation experiments were performed to detect cell proliferation after knocking down G9a. (e) Invasive abilities of shG9a-786-O cells were measured through transwell assay. (f) Migratory abilities of shG9a-786-O cells were measured through wound healing assay (treated for 24 h). (g, h) The expression of protein-related EMT and apoptosis pathway was measured through western blot.

We constructed a stable G9a knockdown RCC line in 786-O using the lentivirus. After the successful establishment of knocking down G9a cell line (Figure 4(a)), it indicated that cell proliferation was inhibited (Figure 4(b)), and cell apoptosis was increased (Figure 4(c)). Moreover, clone formation experiments showed that the clone number was significantly reduced after G9a knocking down (Figure 4(d)). Similarly, the wound healing assay and the transwell assay showed that the knocking down of G9a led to obvious reduction in renal cancer cell migration and invasion, which was consistent with G9a inhibitor treatment (Figures 4(e) and 4(f)). Western blotting showed that the expression of H3K9me2, N-cadherin, vimentin, and Bcl-2 was decreased, and Bax and E-cadherin expression was upregulated (Figures 4(g) and 4(h)). According to these results, the loss of G9a reduced the carcinogenicity of renal cancer. So, we hypothesized that the deletion of G9a caused a decreased H3K9me2 level at the downstream targeted genes, thereby activating their expression and leading to reduced carcinogenicity.

### 3.5. Suppression of SPINK5 Contributes to the Tumor-Promoting Function of G9a

**Figure 5:**
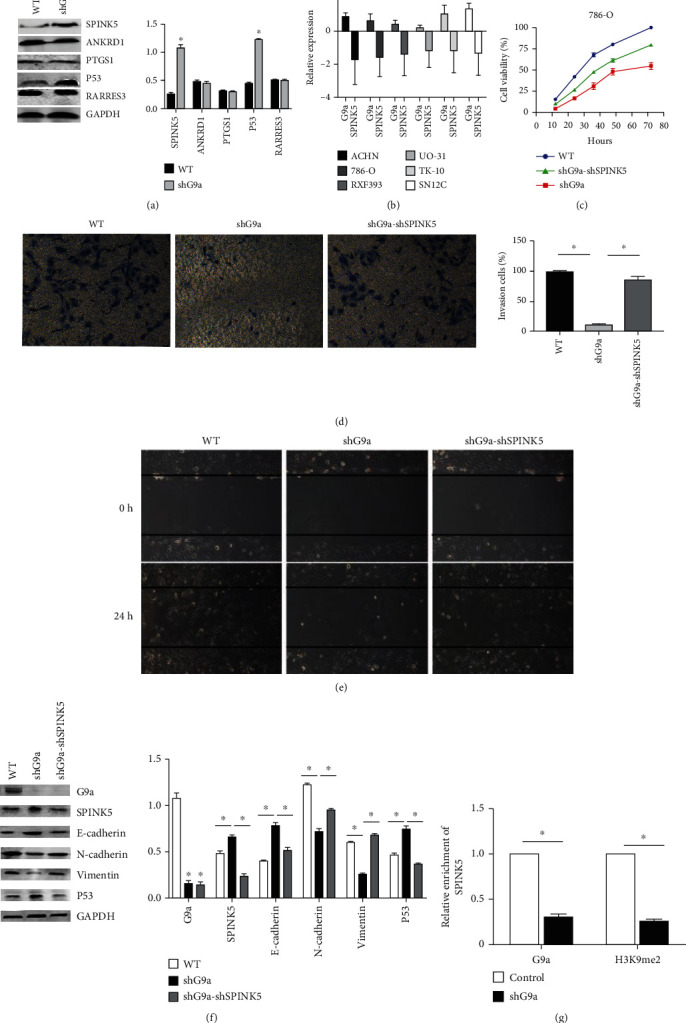
Suppression of SPINK5 contributes to the tumor-promoting function of G9a. (a) The expression of proteins of SPINK5, ANKRD1, PTGS1, P53, and RARRES3 was measured. (b) The expression of G9a was inversely correlated with the expression of SPINK5 in human RCC cell lines though data mining. (c) CCK-8 assay was performed to show the proliferation ability of 786-O after knocking down both G9a and SPINK5 (treated for 12, 24, 36, 48, and 72 h). (d, e) Invasive and migratory abilities of shG9a-shSPINK5-786-O cells were measured through transwell assay and wound healing assay. (f) The expression of protein-related EMT and p53 was measured. (g) ChIP was applied to analyze the interaction of G9a protein (H3K9me2 methyl transferase) and promoter sequence of the downstream target SPINK5. Knockdown of G9a significantly diminished the G9a binding and H3K9me2 level at the promoter region of SPINK5 as determined by ChIP assay.

We hypothesized that G9a could epigenetically silence the expression of the downstream gene. Through a literature review, we identified four genes that might be downstream targets of G9a [25]. We found that only SPINK5 and p53 were upregulated in shG9a-786-O cells (Figure 5(a)). SPINK5 had been shown to inhibit the proliferation, migration, invasion, and migration of esophageal cancer cells via the Wnt/*β*-catenin signaling pathway [26]. Bioinformatics analysis found a correlation between G9a and SPINK5 (Figure 5(b)). The expression of G9a was negatively correlated with the expression of SPINK5 in human RCC cell lines. Through oncomine data mining, it indicated that G9a was upregulated in six RCC cell lines. In contrast, SPINK5 was downregulated in these cell lines. Correlation analysis revealed that the expression of G9a was inversely related to that of SPINK5 in these RCC lines (Figure 5(b)). Therefore, we considered that SPINK5 was one of the downstream genes of G9a. Using shRNA, we knocked down the expression of SPINK5 in the 786-O cell line with stable knockdown of G9a. The results demonstrated that the proliferation, migration, and invasion of 786-O cells were significantly enhanced (Figures 5(c)–5(e)) in G9a and SPRINK5 both knocking down than G9a knocking down only. Finally, G9a could bind to the promoter region of targeted genes. So ChIP assay was applied to analyze the interaction of G9a protein (H3K9me2 methyl transferase) and promoter sequence of SPINK5. A specific primer matching the promoter sequence of SPINK5 was designed and synthesized. The qPCR analysis revealed that, after silencing of G9a via shRNA, the G9a or H3K9me2 binding at the promoter region of SPINK5 was decreased (Figure 5(g)). Also, the results showed that the inhibition of G9a could enhance p53 expression suggesting that p53 might be a downstream target of SPINK5.

### 3.6. G9a Promoted the Proliferation and EMT Process of RCC Cells *In Vivo*

**Figure 6:**
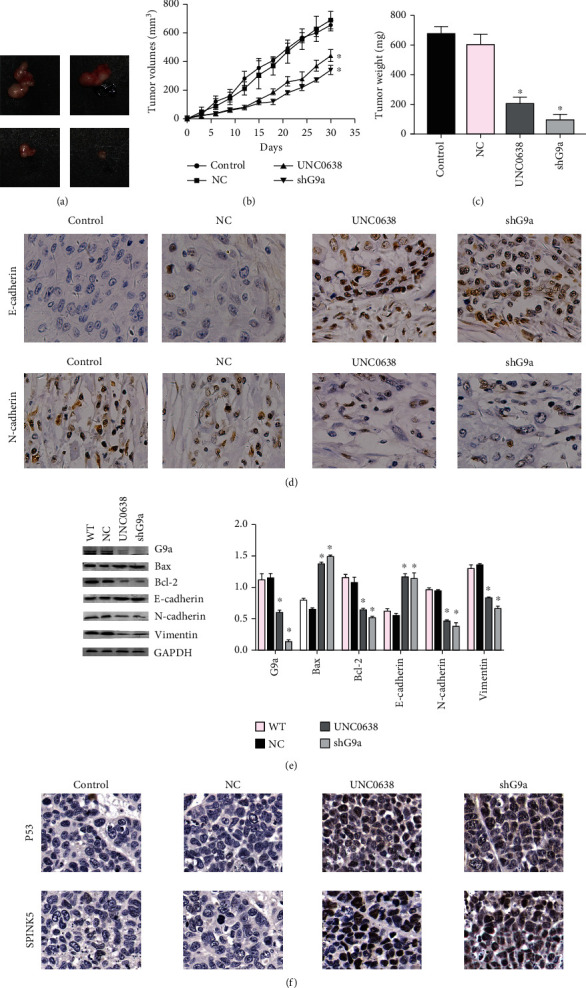
G9a can affect the proliferation and EMT process of RCC cells *in vivo*. (a) Xenograft tumors in each group were harvested and extracted completely after 30 days. (b) Each time point represents mean tumor volume for each group. (c) Tumor weight was obtained at the end of experiment. (d) Immunohistochemical staining was performed to demonstrate that the EMT index of G9a knockdown *in vitro* showed significant changes. (e) The expression of protein-related EMT and apoptosis pathway and G9a was measured by western blot. (f) The expression of proteins P53 and SPINK5 was measured by immunohistochemical staining in tumors.

In Figures 6(a) and 6(b), it was illustrated that, after G9a knocking down or treatment with its inhibitor, the xenograft tumors were significantly smaller than those observed in the control group. In immunohistochemistry (Figure 6(c)), it was found that the expression of N-cadherin was decreased after G9a inhibition, whereas E-cadherin expression increased (Figure 6(d)). Western blotting results also indicated that the expression of N-cadherin, vimentin, and Bcl-2 was decreased after G9a inhibition, whereas Bax and E-cadherin expression was upregulated (Figure 6(e)). Finally, immunohistochemical staining was performed and we found that the P53 and SPINK5 expression was enhanced after G9a inhibition (Figure 6(f)). Therefore, our *in vitro* xenograft experiments demonstrated that G9a could affect the proliferation and EMT processes of RCC cells *in vivo* which were consistent with the results observed in *in vitro* experiments.

## 4. Discussion

This study was aimed at providing evidence to whether G9a has carcinogenic effects and suggested the possible mechanisms that mediate this effect. We first evaluated the effect of G9a using inhibitor UNC0638. The results demonstrated that G9a inhibition inhibits the proliferation with simultaneous induction of apoptosis and inhibits migration and invasion of RCC cells. Then, we used shRNA to prove that loss of G9a causes a decrease in the carcinogenicity of RCC cell lines, and we also demonstrate that SPINK5 is the target gene of G9a. At last, we proved that G9a promoted the proliferation and EMT process of RCC cells in vivo. Therefore, targeting G9a might be a new target to the treatment of kidney cancer.

Cancer is a polygenic disease, and tumorigenesis is related to a variety of factors [27]. Dysfunction of epigenetic regulators, including DNA methylation, histone modifications, and chromatin remodeling, may have a vital impact on the proliferation, apoptosis, migration, and invasion of cancer cells [28, 29]. The expression of G9a is upregulated in various tumors and closely related to poor prognosis [16, 19, 30]. Numerous studies have shown that G9a can inhibit the expression of targeted genes by methylating the H3K9 site on histones [31]. In our study, we demonstrated that the expression of G9a was significantly increased in renal cancer tissues. Moreover, we found that the expression of G9a in TCGA database was closely related to the prognosis of renal cancer. Based on our experimental results, the upregulation of G9a played an important role in the development of human RCC, which was consistent with G9a in other organic cancers. Previous studies showed that G9a achieved carcinogenesis by inhibiting H3K9me2 in the genome of liver cancer cells [25]. Studies indicated that G9a was overexpressed in ovarian cancer and closely related to advanced tumor stage and poor prognosis. After knocking down of G9a, the migratory and invasive abilities of ovarian cancer cells were significantly decreased [17]. As known, G9a could inhibit the expression of tumor suppressor genes to promote tumor progression. In liver cancer, G9a promoted disease progression by silencing the tumor suppressor gene RARRES3 [25]. In lung cancer, the carcinogenicity of G9a was manifested through the inhibition of the epithelial cell adhesion molecule [15]. In breast cancer, G9a could promote tumor invasion by alleviating E-cadherin expression [14]. In addition to its carcinogenic effects in cancer, the role of G9a in transcriptional repression in hypoxic environments has also been demonstrated [32]. Furthermore, G9a exerts a regulatory effect on autophagy and metabolic reprogramming [33, 34]. High expression of G9a increased tumor proliferation, migration, and invasion, whereas it reduces the apoptosis of tumor cells [35]. These results were consistent with the present results. Our study identified a novel downstream gene of G9a, namely, SPINK5. After inhibition or knocking down of G9a, the expression of SPINK5 was significantly increased, demonstrating that G9a could negatively regulate SPINK5, thus promoting tumorigenesis and development.

We selected the G9a-specific inhibitor UN0638 to study the effects of G9a on the RCC cell lines 786-O and SN12C. The results showed that UN0638 exerted an inhibitory effect on the proliferation, migration, and invasion of RCC cells and significantly promoted apoptosis. The western blotting results confirmed this observation, and the expression of H3K9me2 was inhibited after G9a inhibition. The stable shG9a cell line was established with a lentivirus to knock down G9a. The changes noted in the RCC line after G9a knocking down were consistent with those observed in the treatment with the G9a inhibitor. Besides, the knockdown of G9a resulted in the upregulation of the tumor suppressor genes SPINK5 and p53. The anticancer effect of SPINK5 has been recently demonstrated in esophageal cancer, while p53 is a well-known tumor suppressor gene. In order to clarify the relationship of G9a and SPINK5, ChIP assay was performed. The results demonstrated that G9a was binding at SPINK5 gene promoter and inhibits its expression. Then, we examined the mRNA level of P53 and SPINK5 to demonstrate that the change in SPINK5 and p53 protein level is due to transcriptional regulation but not from posttranscriptional regulation. The results showed that the knockdown both of SPINK5 and G9a could enhance the proliferative, migratory, and invasive abilities of tumor cells compared with those observed in G9a knockdown only. Interestingly, the expression level of p53 was consistent with that of SPINK5. Thus, we considered that p53 might be a downstream target of SPINK5. The mechanism of the SPINK family of genes in tumors is currently unclear [36–38]. The expression of SPINK5 in esophageal cancer cells was significantly reduced, and its anticancer effect was very significant. Overexpression of SPINK5 could significantly inhibit the proliferation, migration, and invasion of esophageal cancer cells [26].

## 5. Conclusion

In summary, we demonstrated the carcinogenic effects of G9a using clinical kidney cancer tissues, RCC lines, and animal experiments. G9a was upregulated in renal cancer. It silenced the expression of certain tumor suppressor genes to enhance the proliferation, migration, and invasion and reduces apoptosis of RCC cells *in vitro* and *in vivo*. Furthermore, we identified SPINK5 as the downstream target gene of G9a. Therefore, targeting G9a might be a new target to the treatment of kidney cancer.

## Figures and Tables

**Table 1:** The relationship between G9a expression and patient characteristics.

Parameters	Total	G9a expression		*x* ^2^	*P* value
		High	Low		
	80	45	35
Age					
≥65	44	28	16	2.168	0.14091
<65	36	17	19		
Gender					
Male	47	30	17	2.66	0.1029
Female	33	15	18		
Tumor stage					
I-II	39	17	24	7.472	0.00627
III-IV	41	28	11		
Distant metastasis					
Absence	51	23	28	7.11	0.00767
Presence	29	22	7		

## Data Availability

Data used to support the results of this study can be obtained from the corresponding author.

## Abbreviations

RCC: Renal cell carcinoma
WT: Wild type
NC: Negative control.
